# Responses of Combined Non-starch Polysaccharide Enzymes and Protease on Growth Performance, Meat Quality, and Nutrient Digestibility of Yellow-Feathered Broilers Fed With Diets With Different Crude Protein Levels

**DOI:** 10.3389/fvets.2022.946204

**Published:** 2022-07-18

**Authors:** Chaoyong Wang, Tong Yuan, Jing Yang, Wenxuan Zheng, Qilin Wu, Kaixuan Zhu, Xiangyu Mou, Lizhi Wang, Kangkang Nie, Xinyun Li, Yongwen Zhu

**Affiliations:** ^1^Department of Animal Genetics and Breeding, College of Animal Science and Technology, Huazhong Agricultural University, Wuhan, China; ^2^Guangdong Provincial Key Laboratory of Animal Nutrition and Regulation, College of Animal Science, South China Agricultural University, Guangzhou, China; ^3^Guangdong Guang Ken Animal Husbandry Co., Ltd., Guangzhou, China; ^4^Department of Animal Nutrition and Health, Kemin (China) Technologies Co., Ltd., Zhuhai, China

**Keywords:** non-starch polysaccharide enzymes, protease, nutrients utilization, yellow-feathered broilers, crude protein

## Abstract

The aim of this study was to investigate the responses of non-starch polysaccharide (NSP) enzymes and protease combination on growth performance, meat quality, and nutrients digestibility of yellow-feathered broilers fed with corn-soybean meal basal diets with normal and subnormal crude protein (CP) levels. The experimental design was completely randomized with a 2 × 2 factorial arrangement of treatments, including six replicates of 20 birds per pen. Two basal diets were formulated with normal CP level as positive control (PC) and subnormal CP level without extra essential amino acid (AA) supplementation as negative control (NC). The basal diets were supplemented without or with NSP enzymes and protease. Broilers fed with the NC diet had lower (*P* < 0.05) final body weight (BW), average daily weight gain (ADG) on days 1–21, 22–56 and 1–56 and higher (*P* < 0.05) feed-to-gain ratio (F/G) on day 22–56 than those fed with PC diet. The broilers fed with the NC diet had higher (*P* < 0.05) L^*^ and b^*^ values in thigh muscle, crypt depth in the duodenum, and dry matter (DM) digestibility as well as lower (*P* < 0.05) villus height, musculature thicknesses, and villus height: crypt depth in the duodenum than those fed with the PC diet. Dietary NSP enzymes and protease combination increased (*P* < 0.05) the ADG and F/G of the broilers on days 1–56, and pH values in breast and thigh muscles as well as the digestibility of DM, gross energy (GE), CP and most AAs of the broilers on day 56. Compared with the PC diet, the combination of NSP enzymes and protease exhibited greater (*P* < 0.05) improvements in the digestibility of DM, CP, and some AAs (Asp, Ile, and Leu) in the broilers fed with the NC diet. In conclusion, reducing CP diet without essential AA supplementation impaired the growth performance and meat color of the thigh muscles of the broilers. The combination of NSP enzymes and protease effectively improved the growth performance, meat quality, and nutritional values of the broilers. In terms of the digestibility of DM, CP, and some AAs, the magnitude of response to the addition of NSP enzymes and protease was greater in the low nutritional-quality diet with a subnormal CP level.

## Introduction

With the increasing demand and price volatility of ingredients, a concerted effort to improve the nutritive value of poultry feeds with exogenous enzyme supplementation has been ongoing in the poultry industry (1, 2). The existing knowledge is that exogenous enzymes could play a great role in enhancing the potential feeding value of poultry diets (3–5). In addition to the use of individual exogenous enzymes, combinations of exogenous enzyme preparations (i.e., carbohydrase, phytase, and protease) have also been proposed to be more effective in enhancing growth performance and nutrient availability in poultry (6–8). A number of studies have demonstrated that a combination of exogenous non-starch polysaccharide (NSP) enzymes and protease supplementation was used expectantly to improve growth performance and nutrient ultilization, and reduce nutrient excretion in broilers (9) and hens (7, 10). The efficiency of exogenous enzyme combinations varied depending on dietary nutritional quality and substrate specificity (8, 11). Responses to exogenous enzyme additions were expected to be greater in diets the lower ingredient quality and nutritional density (12–14). The multi-enzyme addition of xylanase, amylase, and protease exhibited greater effects on increasing energy digestibility of corn-soybean meal diets with lower energy contents compared with diets with higher energy levels in broiler chickens (15). However, several studies have indicated that there was no beneficial responses of exogenous enzyme supplementation on performance and nutrient digestibility of broiler chickens fed WITH diets varying in nutrient density (16, 17). The lack of effect of dietary exogenous enzymes could be that a greater reduction in the nutritional levels of diets could have possibly elicited the beneficial effects of enzyme supplementations (16, 18). So far, research data on possible interactions between dietary crude protein (CP) levels and exogenous enzyme supplementation in broiler diets were not only limited but also inconsistent and as a result, more information is still required. Therefore, this study was conducted to investigate whether there were different responses of combined NSP enzymes and protease supplementation on growth performance, carcass traits, meat quality and nutrient utilization in broiler chickens fed diets with normal and subnormal CP levels.

## Methods and Materials

### Experimental Design and Diets

The study procedures were reviewed and approved by the Institutional Animal Care and Use Committee of South China Agricultural University to ensure compliance with welfare and humane practices (SCAU-10564)). The experimental design was completely randomized with a 2 × 2 factorial arrangement of treatments. Two basal diets were formulated with normal CP level as positive control (PC) and subnormal CP level without extra essential amino acid (AA) supplementation as negative control (NC). The basal diets were supplemented without (control) or with exogenous NSP enzymes and protease combination (enzyme). The NSP enzymes (Kemzyme^®^ FHS, enzyme activities: xylanase 80,000 U/g, and β-glucanase 6,000 U/g) and protease (Kemzyme^®^, heat- and acid-resistant protected protease, activity: 8,000 U/g) were provided by Kemin (China) Technologies Co., Ltd. (Zhuhai, Guangdong, China). The recommended level of 200 mg/kg enzyme mixtures was added to the diets at the expense of corn (14), providing 16,800 U of xylanase, 1,180 U of β-glucanase, and 1,530 U of protease per kg diet based on the analysis.

A total of 480-day-old male yellow-feathered broilers were kept in floor pens in a building with a controlled environment. The birds were weighed individually and divided into four dietary treatments with 6 replicate pens of 20 birds per pen. The room temperature was initially set at 34°C and was accordingly reduced by 2°C per week to maintain 20°C. A lighting program of 18-h light and 6-h darkness was maintained throughout the trial except for the first day when the birds had 24 h of light. The PC diet was formulated to meet the nutrient requirements of the National Agricultural Industry Standard (NY/T 3645-2020) for yellow-feathered broilers and contained CP levels of 22 and 19.5% for broilers at the starter and grower-finisher phases, respectively (Table 1). The NC diets were formulated with subnormal CP levels of 20 and 18% without extra essential AA supplementation compared to the PC diet, while other nutrients met the requirements for broilers in the two phases. Both the PC and NC diets were without or with supplemental exogenous enzymes according to the experimental design. Each diet contained 5 g/kg titanium dioxide (TiO_2_) as an indigestible marker for calculation of nutrient digestibility coefficients (19). The diets were pelleted using a conditioning temperature of 65°C. All the diets were fed in a pelleted form throughout the experimental period. The experimental diets and water were provided *ad libitum*.

**Table 1:** Composition and nutrient levels of the positive and negative control diets.

**Item**	**Starter period (days 1–21)**		**Grower-finisher period (days 22–56)**	
	**PC**	**NC**	**PC**	**NC**
**Ingredient, %**
Corn	55.28	62.18	56.37	61.54
Soybean meal	29.3	23.38	26.57	22.2
Corn gluten meal	8.2	8.2	6.0	6.0
Lard oil	2.08	1.1	7.0	6.2
Limestone	1.06	1.06	1.2	1.2
Dicalcium phosphate	2.0	2.0	1.1	1.1
L-lysine·HCl	0.41	0.41	0.2	0.2
DL-Methionine	0.11	0.11	0.06	0.06
Choline chloride	0.1	0.1	0.08	0.08
Sodium bicarbonate	0.26	0.26	0.24	0.24
Sodium chloride	0.20	0.20	0.18	0.18
Vitamin-mineral premix^1^	1.0	1.0	1.0	1.0
Total	100	100	100	100
**Calculated value, %**
Metabolizable energy, Kcal/kg	2,950	2,950	3,253	3,251
Crude protein^2^	22.03	20.02	19.51	18.03
Calcium	0.95	0.94	0.8	0.79
Total phosphate	0.71	0.69	0.53	0.52
Lysine	1.15	1.02	0.95	0.86
Methionine	0.50	0.47	0.40	0.38
Methionine + cysteine	0.90	0.86	0.70	0.67
Threonine	0.85	0.80	0.67	0.62
Tryptophan	0.20	0.17	0.16	0.14

*^1^Premix provided the following per kilogram of diets for the starter period: VA 12,000 IU, VD_3_ 600 IU, VE 45 IU, VK 2.5 mg, VB_1_ 2.4 mg, VB_2_ 5 mg, VB_6_ 3.5 mg, VB_12_.01 mg, niacin 42 mg, D-calcium pantothenate 10 mg, folic acid 1 mg, biotin.15 mg, Fe 80 mg, Cu 8 mg, Mn 80 mg, Zn 85 mg, I.7 mg, Se.15 mg; Premix provided the following per kilogram of diets for the grower-finisher period: VA 9,000 IU, VD_3_ 500 IU, VE 35 IU, VK 2.2 mg, VB_1_ 2.4 mg, VB_2_ 5 mg, VB_6_ 6 mg, VB_12_.07 mg, niacin 35 mg, D-calcium pantothenate 18 mg, folic acid.7 mg, biotin.1 mg, Fe 80 mg, Cu 7 mg, Mn 60 mg, Zn 80 mg, I 0.60 mg, and Se.15 mg*.

*^2^Analyzed values based on triplicate determinations*.

*PC, positive control; NC, negative control*.

At 21 and 56 days of age, after 12-h feed withdrawal, the birds were weighed, and feed consumption was recorded by each replicate pen. Average daily gain (ADG), average daily feed intake (ADFI), and feed:gain ratio (F:G) were calculated following any necessary corrections for mortality. Mortality was very low and averaged.95% throughout the experimental period. On day 56, based on the average body weight (BW) of birds in each replicate pen, two birds per each replicate were selected and euthanized by CO_2_ inhalation, and then killed by bleeding. The left breast and thigh meats were removed and weighed to determine the percentages of breast and thigh meats relative to live BW at processing. The breast and thigh muscles were sliced and weighed. The concentration of hydrogen ion was estimated using a microprocessor pH meter (model pH 211; Hanna Instruments, Woonsocket, RI), which was set into incisions on the cranial left side of the muscles. Two measurements were recorded, and the mean pH value of the muscles of each carcass was calculated. Hunter L^*^ (lightness), a^*^ (redness), and b^*^ (yellowness), of the meat were measured using a Minolta CR410 chromameter (Konica Minolta Sensing, Osaka, Japan) in two different fields of the internal face of the cranial position of the post-mortem. Segments of about 1.5 cm from the middle of the duodenum and jejunum were excised and flushed with ice-cold saline and immediately placed in 4% paraformaldehyde for morphometric analysis. The indices of villus height, crypt depth, and muscular thickness were measured by computer-aided light microscope image analysis. Excreta samples were collected per cage over three consecutive days (from days 54 to 56) for determination of nutrient digestibility. Multiple subsamples from three consecutive days were pooled, homogenized, and stored in airtight containers at 4°C until further analysis. Diets and excreta samples were analyzed for DM, GE, CP, and AAs.

### Sample Analyses

Measurement of DM was performed according to the Association of Official Analytical Chemists standard procedures (AOAC 930.15; AOAC, 2007) at 135°C for 2 h. GE values were measured using an isoperibol oxygen bomb calorimeter (Kalorimeter C7000 prozesso, IKA, Staufen, Germany). Determination of CP content was performed with the Kjeldahl method (method 984.13; AOAC, 2007) on a Kjeltec TM 8400 apparatus (FOSS Inc., Eden Prairie, MN, United States). One xylanase unit was defined as the amount of enzyme that releases.48 μmol of reducing sugar as xylose from wheat arabino xylan per minute at pH 4.2 and 50°C. One protease unit was defined as the amount of enzyme that releases 1 μg of phenolic compound, expressed as tyrosine equivalents, from a casein substrate per minute at pH 7.5 and 40°C. TiO_2_ concentrations were determined in triplicate for diets and digesta samples, respectively, with the colorimetric method. The nutrient digestibility of DM, GE, CP, and AAs of the diets were calculated according to the following formulas:


$$\begin{array}{r} \text{ Nutrient digestibility }\left(\%\right)=\\ \left\{\text{1}-\left[\left({\text{TiO}}_{\text{2 feed}}/{\text{TiO}}_{\text{2 excreta}}\right)\times \left({\text{Nutrient}}_{\text{excreta}}/{\text{Nutrient}}_{\text{feed}}\right)\right]\right\}\times \text{1}00. \end{array}$$

### Statistical Analyses

All the data were analyzed by two-way ANOVA using the general linear model procedure of SAS 9.2 (SAS Institute, 2010). The model included the main effects of dietary CP levels, dietary enzyme treatments, and their interactions. Either one replicate cage or one broiler served as an experimental unit, and the values presented in the Tables are means with pooled standard error of mean (SEM). Differences among the means were tested with the LSD method, and statistical significance was set at *P* < 0.05.

## Results

### Growth Performance and Carcass Traits

The results of growth performance and carcass traits are shown in Tables 2, 3. The NC group had lower (*P* < 0.05) final BW and ADG of broilers on days 1–21, 22–56, and 1–56 and higher (*P* < 0.05) F/G on days 22–56 than the PC group. Compared with the control group, the combination of dietary NSP enzymes and protease increased (*P* < 0.05) the final BW and ADG of broilers on days 1–21, 22–56, and 1–56 but decreased (*P* < 0.05) the F/G (*P* < 0.05) on days 22–56. Dietary CP levels and enzyme supplementation had no effect (*P* > 0.05) on ADFI on days 1–21, 22–56, and 1–56 and on the percentage of dressing, eviscerated yield, breast muscle, thigh muscle, and abdominal fat on day 56. There were no significant interactions (*P* > 0.05) between dietary CP levels and enzyme supplementation in all the above-mentioned indices.

**Table 2:** Effects of NSP enzymes and protease on growth performance of yellow-feathered broilers fed with diets with different CP levels during days 1–56.

**Item**	**PC** ^**1**^		**NC** ^**1**^		**SEM**	**Dietary CP** ^**2**^		**SEM**	**Enzyme treatment** ^**2, 3**^		**SEM**	***P*** **-value**		
	**Control**	**Enzyme**	**Control**	**Enzyme**		**PC**	**NC**		**Control**	**Enzyme**		**CP**	**Enzyme**	**CP × enzyme**
**Days 1–21**
BW, g/bird	414.5	431.1	407.5	424.3	2.85	422.8^a^	415.9^b^	2.01	411^b^	427.7^a^	2.01	0.03	<0.0001	0.97
ADG, g/bird/day	19.8	19.9	18.7	19.6	0.14	19.9^a^	19.1^b^	0.10	19.2^b^	19.7^a^	0.10	0.01	<0.0001	0.86
ADFI, g/bird/day	43.6	41.65	42.3	41.4	1.09	42.63	41.84	0.77	42.9	41.5	0.77	0.48	0.21	0.63
F/G	2.20	2.09	2.26	2.12	0.06	2.19	2.19	0.04	2.28^a^	2.11^b^	0.04	0.95	0.007	0.68
**Days 22–56**
BW, g/bird	2,405	2,489	2,361	2,401	14.0	2,447^a^	2,381^b^	9.92	2,383^b^	2,445^a^	9.90	0.0001	0.0003	0.14
ADG, g/bird/day	56.9	58.8	55.8	56.5	0.41	57.8^a^	56.1^b^	0.29	56.3^b^	57.6^a^	0.29	0.0005	0.005	0.14
ADFI, g/bird/day	130.5	129.2	132.4	128.5	1.93	129.9	130.5	1.36	131.4	128.8	1.36	0.76	0.19	0.53
F/G	2.29	2.20	2.37	2.28	0.035	2.25^b^	2.32^a^	0.025	2.33^a^	2.23^b^	0.03	0.04	0.01	0.96
**Days 1–56**
ADG, g/bird/day	43.1	44.7	42.3	43.1	0.25	43.9^a^	42.7^b^	0.18	42.7^b^	43.9^a^	0.17	0.001	0.002	0.14
ADFI, g/bird/day	97.9	96.4	98.6	95.8	1.51	97.15	97.2	1.07	98.3^a^	96.1^b^	1.07	0.96	0.17	0.71
F/G	2.27	2.16	2.33	2.23	0.035	2.22	2.28	0.025	2.30^a^	2.20^b^	0.03	0.08	0.007	0.91

*^a, b^Within a column, means without a common superscript differ (P < 0.05)*.

*^1, 2, 3^Data represent the means of 6 and 12 replicates (n = 6 and 12)*.

*PC, positive control with normal CP level; NC, negative control with subnormal CP level; ADFI, average daily feed intake; ADG, average daily gain; BW, body weight; F:G, feed:gain ratio; for enzyme treatment, the basal diets were supplemented without or with a combination of NSP enzymes and protease supplementation that was supplied with 16,000 U of xylanase, 1,200 U of β-glucanase, and 1,600 U of protease per kg diet*.

**Table 3:** Effects of NSP enzymes and protease on carcass traits of yellow-feathered broilers fed with diets with different CP levels on day 56.

**Item, %**	**PC** ^**1**^		**NC** ^**1**^		**SEM**	**Dietary CP** ^**2**^		**SEM**	**Enzyme treatment** ^**2, 3**^		**SEM**	***P*** **-value**		
	**Control**	**Enzyme**	**Control**	**Enzyme**		**PC**	**NC**		**Control**	**Enzyme**		**CP**	**Enzyme**	**CP × enzyme**
Dressing	76.7	75.69	75.64	76.54	0.97	76.20	76.09	0.69	76.17	76.12	0.69	0.91	0.95	0.34
Eviscerated yield	62.02	62.08	62.11	62.24	1.05	62.05	62.175	0.74	62.07	62.16	0.74	0.72	0.78	0.62
Breast muscle	14.87	13.90	13.27	13.52	0.94	14.39	13.395	0.72	14.07	13.71	0.72	0.33	0.55	0.41
Thigh muscle	19.1	19.36	18.97	17.49	1.02	19.23	18.23	0.69	19.04	18.43	0.67	0.30	0.71	0.53
Abdominal fat	2.98	2.54	2.21	2.32	0.41	2.76	2.265	0.29	2.60	2.43	0.24	0.24	0.68	0.51

*^1, 2, 3^Data represent the means of 6 and 12 replicates (n = 6 and 12)*.

*PC, positive control with normal CP level; NC, negative control with subnormal CP level; for enzyme treatment, the basal diets were supplemented without or with a combination of NSP enzymes and protease supplementation that was supplied with 16,000 U of xylanase, 1,200 U of β-glucanase, and 1,600 U of protease per kg diet*.

### Meat Quality

The results of meat quality are shown in Table 4. The broilers fed with the NC diet had higher (*P* < 0.05) L^*^ and b^*^ values in the thigh muscle of the broilers on day 56 than those fed the PC diet. Dietary NSP enzyme and protease supplementation increased (*P* < 0.05) pH values and had no effect (*P* > 0.05) on L^*^ and b^*^ values in in both breast and thigh muscles of the broilers on day 56. There were no significant interactions (*P* > 0.05) between dietary CP levels and enzyme supplementation in the measured indices related to meat quality.

**Table 4:** Effects of NSP enzymes and protease on meat quality of yellow-feathered broilers fed with diets with different CP levels on day 56.

**Item**	**PC** ^**1**^		**NC** ^**1**^		**SEM**	**Dietary CP** ^**2**^		**SEM**	**Enzyme treatment** ^**2, 3**^		**SEM**	***P*** **-value**		
	**Control**	**Enzyme**	**Control**	**Enzyme**		**PC**	**NC**		**Control**	**Enzyme**		**CP**	**Enzyme**	**CP × enzyme**
**Breast muscle**
L*	55.52	56.1	55.5	52.6	1.83	55.8	54.1	0.74	55.5	54.4	1.29	0.36	0.52	0.35
a*	8.37	8.81	8.24	7.61	0.81	8.59	7.93	0.72	8.31	8.21	0.57	0.42	0.9	0.52
b*	2.69	2.64	2.42	2.72	0.76	2.66	2.57	0.69	2.56	2.68	0.53	0.90	0.87	0.81
pH	6.75	7.20	6.62	7.12	0.08	6.975	6.87	0.29	6.69^b^	7.16^a^	0.06	0.24	<0.0001	0.80
**Thigh muscle**
L*	54.9	56.0	59.6	58.1	1.44	55.49^b^	58.83^a^	0.89	57.3	57.1	0.89	0.01	0.87	0.33
a*	9.16	9.82	11.77	10.29	0.71	9.49	11.03	0.47	10.47	10.06	0.47	0.15	0.95	0.97
b*	1.97	2.11	2.93	2.04	0.67	2.04^b^	2.49^a^	0.50	2.45	2.08	0.50	0.04	0.57	0.15
pH	6.87	6.97	6.72	7.07	0.08	6.92	6.90	0.06	6.80^b^	7.02^a^	0.06	0.75	0.01	0.18

*^a, b^Within a column, means without a common superscript differ (P < 0.05)*.

*^1, 2, 3^Data represent the means of 6 and 12 replicates (n = 6 and 12)*.

PC, positive control with normal CP level; NC, negative control with subnormal CP level; L^*^, lightness; a^*^, redness; b^*^, yellowness; for enzyme treatment, the basal diets were supplemented without or with a combination of NSP enzymes and protease supplementation that was supplied with 16,000 U of xylanase, 1,200 U of β-glucanase, and 1,600 U of protease per kg diet.

### Intestinal Histomorphology

The results of intestinal histomorphology are shown in Table 5. Broilers fed with the NC diet had lower (*P* < 0.05) villus height, musculature thicknesses, villus height: crypt depth, and higher crypt depth (*P* < 0.05) in the duodenum of broilers on day 56 than those fed with the PC diet. The broilers fed with the NC diet had lower (*P* < 0.05) musculature thickness in the jejunum on day 56 than those fed with the PC diet. Dietary CP level had no effect (*P* > 0.05) on villus height, crypt depth, and villus height:crypt depth in the jejunum. Dietary enzyme supplementation and interactions between dietary CP levels and enzymes supplementation (*P* > 0.05) did not influence the measured indices of intestinal histomorphology.

**Table 5:** Effects of NSP enzymes and protease on intestinal histomorphology of yellow-feathered broilers fed with diets with different CP levels on day 56.

**Item, μm**	**PC** ^**1**^		**NC** ^**1**^		**SEM**	**Dietary CP** ^**2**^		**SEM**	**Enzyme treatment** ^**2, 3**^		**SEM**	***P*** **-value**		
	**Control**	**Enzyme**	**Control**	**Enzyme**		**PC**	**NC**		**Control**	**Enzyme**		**CP**	**Enzyme**	**CP × enzyme**
**Duodenum**
Villus height	887	879	723	814	50	883^a^	769^b^	35	805	847	35	0.03	0.41	0.33
Crypt depth	146	154	174	172	9.9	150^b^	173^a^	7	160	163	7	0.03	0.75	0.62
Musculature thicknesses	174	190	152	151	14.3	182^a^	152^b^	10	163	171	10	0.04	0.63	0.56
Villus height: Crypt depth	6.10	5.93	4.22	4.74	0.42	6.02^a^	4.48^b^	0.29	5.16	5.34	0.29	0.001	0.69	0.42
**Jejunum**
Villus height	952	911	907	969	45	932	938	33	930	940	33	0.95	0.87	0.29
Crypt depth	155	153	167	162	13.2	154	165	10	161	158	10	0.41	0.79	0.92
Musculature thicknesses	198	196	178	155	0.67	197^a^	167^b^	14	188	176	14	0.04	0.42	0.48
Villus height: Crypt depth	6.37	6.10	5.47	6.07	0.43	6.24	5.77	0.31	5.92	6.09	0.31	0.27	0.74	0.33

*^a, b^Within a column, means without a common superscript differ (P < 0.05)*.

*^1, 2, 3^Data represent the means of 6 and 12 replicates (n = 6 and 12)*.

*PC, positive control with normal CP level; NC, negative control with subnormal CP level; for enzyme treatment, the basal diets were supplemented without or with a combination of NSP enzymes and protease supplementation that was supplied with 16,000 U of xylanase, 1,200 U of β-glucanase, and 1,600 U of protease per kg diet*.

### Nutrient Utilization

The results of nutrient utilization are shown in Table 6. Dietary CP levels affected (*P* < 0.05) DM digestibility but had no effect (*P* > 0.05) on the digestibility of GE and CP. Dietary enzyme supplementation influenced (*P* < 0.05) the digestibility of DM, GE, CP, and most AAs. There were significant interactions (*P* < 0.05) between dietary CP levels and enzyme in digestibility of DM, CP, and some AAs (Asp, Ile, and Leu). The broilers fed with the NC diet had higher (*P* < 0.05) DM digestibility than those fed with the PC diet. The combination of dietary NSP enzyme and protease increased (*P* < 0.05) the digestibility of DM, GE, CP, and most AAs. Dietary enzyme supplementation exhibited greater improvements in the digestibility of DM, CP, and some AAs (Asp, Ile, and Leu) of broilers in NC group than in the PC group. No significant interactions (*P* > 0.05) between dietary CP levels and enzyme supplementation were observed in other indices mentioned above.

**Table 6:** Effects of NSP enzymes and protease on digestibility of DM, GE, CP, and amino acids of yellow-feathered broilers fed with diets with different CP levels.

**Item, %**	**PC** ^**1**^		**NC** ^**1**^		**SEM**	**Dietary CP** ^**2**^		**SEM**	**Enzyme treatment** ^**2, 3**^		**SEM**	***P*** **-value**		
	**Control**	**Enzyme**	**Control**	**Enzyme**		**PC**	**NC**		**Control**	**Enzyme**		**CP**	**Enzyme**	**CP × enzyme**
DM	73.3^bc^	74.7^a^	71.0^c^	74.1^a^	0.32	74.0	72.5	0.69	72.2	74.4	0.69	0.0001	<0.0001	0.003
GE	76.4	79.8	76.2	80.0	0.24	78.1	78.1	0.74	76.3^b^	79.9^a^	0.74	0.87	<0.0001	0.36
CP	50.8^b^	53.0^a^	49.7^c^	53.5^a^	0.39	51.9	51.6	0.72	50.3	53.3	0.72	0.42	<0.0001	0.05
Asp	53.3^bc^	56.2^a^	52.1^c^	57.7^a^	1.16	54.8	54.9	0.82	52.7	56.9	0.82	0.35	<0.05	0.04
Thr	57.1	62.2	57.2	64.0	1.48	59.6	60.6	0.60	57.1^b^	63.1^a^	0.60	0.47	0.0002	0.41
Ser	58.2	56.5	57.1	59.9	0.92	57.4	58.5	0.78	57.7^b^	58.2^a^	0.77	0.78	0.0006	0.59
Glu	56.9	60.0	57.4	60.3	1.78	58.4	58.9	1.06	57.2^b^	60.1^a^	1.06	0.66	0.004	0.46
Ala	48.2	53.5	49.1	52.9	1.45	50.8	51.0	0.95	48.6^b^	53.2^a^	0.95	0.58	<0.0001	0.41
Val	57.6	59.2	58.3	60.4	2.29	58.4	59.4	1.58	58.0^b^	59.8^a^	1.58	0.92	<0.0001	0.52
Ile	52.5^c^	56.3^b^	53.7^c^	59.4^a^	1.30	54.4	56.6	0.63	53.1	57.9	0.63	0.74	<0.0001	0.01
Leu	59.9^b^	62.0^a^	56.5^c^	62.4^a^	1.80	60.9	59.5	1.28	58.2	62.2	1.28	0.002	0.03	0.001
Tyr	64.8	67.6	62.7	66.3	1.59	66.2^a^	64.5^b^	0.84	63.7^b^	66.9^a^	0.84	0.00	0.00	0.82
Phe	59.4	59.2	63.1	64.6	1.81	59.3	63.9	0.99	61.3	61.9	0.99	0.72	0.56	0.76
Lys	56.4	60.0	55.5	58.7	2.37	58.2	57.1	1.38	56.0^b^	59.3^a^	1.38	0.19	0.00	0.70
His	59.8	57.4	59.0	59.6	2.91	58.6	59.3	1.77	59.4	58.5	1.77	0.49	0.62	0.46
Arg	57.1	62.3	59.4	64.6	1.64	59.7	62.0	1.07	58.3^b^	63.4^a^	1.08	0.38	0.00	0.85
Pro	61.2	64.6	60.9	63.7	1.28	62.9	62.3	0.81	61.0^b^	64.2^a^	0.81	0.41	<0.0001	0.52

*^a, b, c^Within a column, means without a common superscript differ (P < 0.05)*.

*^1, 2, 3^Data represent the means of 6 and 12 replicates (n = 6 and 12)*.

*PC, positive control with normal CP level; NC, negative control with subnormal CP level; CP, crude protein; GE, gross energy; for enzyme treatment, the basal diets were supplemented without or witha combination of NSP enzymes and protease supplementation that was supplied with 16,000 U of xylanase, 1,200 U of β-glucanase, and 1,600 U of protease per kg diet*.

## Discussion

The efficacy of exogenous enzymes in poultry has been previously reported, and there was considerable evidence to support their beneficial effects on poultry productivity (2, 20). In the current study, the relative responses of feed exogenous enzymes were assessed in yellow-feathered broilers fed with diets with different dietary CP levels, namely, normal and suboptimal CP levels. Several studies have suggested that reducing CP levels in diets with adequate supplementation of indispensable amino acids could not completely compromise the growth performance of broilers (21–23). The NC diet was expected to decrease the final BW and ADG on days 1–21, 22–56, and 1–56 compared to the PC diet. When the broilers consumed a feed intake similar to the two different CP diets, birds fed with the NC diet had lower total CP and essential AA intake than those fed with the PC diet, and the poor performance could be attributed to possible deficiency in essential AAs. In addition, the broilers fed with the NC diet had greater F/G than those fed with the PC diet on days 22–56, but no differences in F/G were observed between PC and NC diets on days 1–21 and 1–56. The different aged responses to the nutritional levels of diets were agreed with previous study (24). One explanation was that endogenous enzymatic activities and the microbial community in the digestive tract might not be completely developed in the early stage, which could be due to lack of significant growth response of broilers fed with diets with different nutritional densities (18, 25). In the current study, a combination of NSP enzymes and protease supplementation significantly improved the growth performance of broilers fed with both the PC and NC diets regardless of CP levels on days 1–21, 22–56, and 1–56. The positive results were in agreement with those reported in chick broilers (7) and ducks (26). However, they were inconsistent with studies that reported that mixture enzyme additions had no impact or negative impact on performance variables (17, 27). The varied effects of enzyme treatments on growth performance could depend on enzymes sources and activities, dietary compositions, and specific substrates and age of birds. The nutritional quality of a diet was probably an important factor that influenced the efficiency of an enzyme product. Responses to exogenous enzyme product additions were expected to be greater in diets with lower nutritional quality (12–14). In the present study, no interaction between dietary CP level and supplemental enzyme mixture level was observed in growth performance. One explanation could be that the extent of CP reduction in the NC diet could have possibly elicited the greater responses of enzyme addition to promote growth.

The carcass traits results in the current study indicated that lowering the CP levels in diets had no effect on the relative percentage of breast and thigh muscles and abdominal fat of yellow-feathered broilers, which was similar to the previous finding (28). However, other studies have shown a significant increase in abdominal fat deposition when broilers or ducks were fed with low-CP diets (29, 30). Our study demonstrated that the light and yellow colors of thigh muscles were influenced by dietary CP levels. The greater amount of meat light color reflected a greater degree of protein denaturation and a lower heme pigment concentration in broilers fed with NC diets. Twenty-hours after slaughtering, supplemental dietary enzymes increased the pH values of breast and thigh muscles in broilers on day 56, which was in association with decreased drip loss of the meat. In contrast, other authors have reported that commercial enzyme supplementation was less effective in improving meat quality parameters of broiler chickens (31, 32). The effects of enzyme treatments were inconsistent and varied because of pre-slaughter responses to stress, storage time and temperature, and glycogen reserves at slaughter. Therefore, further studies are needed to determine the effects of enzyme supplementation on the meat quality of broiler chickens.

It is well-documented that exogenous enzyme products could play great role in enhancing the feeding value of poultry diets with different nutritional qualities (3, 5). In the present study, broilers given the NC diet had lower DM digestibility than birds given the PC diet, but there was no effect on GE and CP digestibility. The decreased DM digestibility could be attributed to the lower villus height and villus height:crypt depth ratio of the duodenum in broilers fed with the NC diet. However, dietary nutrient density had no effect on the digestibility of nitrogen, calcium, phosphorus, and most amino acids in the broilers (12). The combination of NSP enzymes and protease supplementation was effective in increasing the digestibility of DM, GE, CP, and most AA of the corn-soybean basal diet used in our study, attributing to chick growth more efficiently (33). The beneficial effects noted in the current study were in agreement with previous studies (33). The NSP content of feeds could impair nutrient digestibility, both directly because of physical hindrance and indirectly because of physiological changes in the gut such as increased digesta viscosity (26). Xylanase and glucanase could degrade cell wall structures and then release encapsulated nutrients most likely contributing to overall improvements in nutrient utilization by reducing the digesta viscosity and increasing the accessibility to digestive enzymes. Then, protease contributed to the hydrolysis of large protein molecules into more absorbable peptides and AAs, enhancing the overall digestion and absorption of CP and AAs (34). Additionally, the beneficial effects on CP and AA digestibility could be mediated by reduction in secretion of amino acid-rich endogenous proteins. Moreover, exogenous NSP enzymes and protease could work together to provide a proper condition for the action of exogenous amylase on starch, which can result in increased DM and energy digestibility (13). Interestingly, there was no effect of enzyme supplementation on feed intake and intestinal histomorphology of the duodenum and jejunum, suggesting that the observed performance responses were due to changes in the digestibility of energy and CP rather than nutrient intake. These findings were in accordance with those who also reported no effect of addition of a mixture of enzymes on the digestibility of energy and CP in the starter phase of broiler chickens (18, 27). The inconsistent and varied effects of enzyme products depended on dietary nutritional composition and levels, enzyme product profile, and age of the birds. Responses to exogenous enzyme addition were expected to be greater in a diet with low-nutrient density than one that provided higher nutrient density to the animals at or above requirements (12–14). Previous study have showed that the combined addition of pectinase, protease, and amylase significantly improved AMEn when added to a corn soybean meal basal diet with lower energy and protein levels (16, 17). In the current study, an interaction between dietary CP level and enzymes was present for the digestibility of DM, CP, and some AAs (Asp, Ile, and Leu). The increased digestibility of DM, CP, and some AAs (Asp, Ile, and Leu) by supplemental enzymes was more apparent in birds fed with the NC diet with reduced CP concentration. It is noteworthy that DM and Leu digestibility of birds fed with the enzyme-supplemented NC diet was restored to the similar level in birds fed with the PC diet. The different responses of enzymes to dietary CP levels offered more flexibility in the use of enzymes for corn-soybean-based meal diets to reduce feed cost in poultry production.

In conclusion, the dietary subnormal CP level without essential AA supplementation impaired the growth performance and meat color of the thigh muscle of yellow-feathered broilers on day 56. The combination of NSP enzymes and protease supplementation effectively improved growth performance, pH value of muscle meat, and the nutritional value of a corn-soybean-based meal diet of yellow-feathered broilers. The magnitude of the responses to exogenous enzyme addition was greater in diets with subnormal nutritional CP level in terms of the digestibility of DM, CP, and some AAs.

## Data Availability Statement

The original contributions presented in the study are included in the article/supplementary material, further inquiries can be directed to the corresponding author/s.

## Ethics Statement

The animal study was reviewed and approved by South China Agricultural University (SCAU-10564). Written informed consent was obtained from the owners for the participation of their animals in this study.

## Author Contributions

CW and TY conceived and designed research. JY, WZ, and QW was responsible for data analysis and interpretation. KZ and XM interpreted the results. LW and KN was responsible for manuscript revision. YZ and XL wrote the paper. All authors contributed to the article and approved the submitted version.

## Funding

This study was sponsored by the Provincial Natural Science Foundation for Co-operation with WENS Group (2019B1515210031), Kemin Animal Nutrition and Health China (2020-TS-NE-B03), Guangdong Provincial Science and Technology Special Foundation (210723106900762 and 2021020103-2), and WENS Group Co-operation Research Project (WENS-2021-KCZX-004).

## Conflict of Interest

JY was employed by Guangdong Guang Ken Animal Husbandry Co., Ltd., China and LW and KN were employed by Kemin (China) Technologies Co., Ltd., China. The remaining authors declare that the research was conducted in the absence of any commercial or financial relationships that could be construed as a potential conflict of interest.

## Publisher's Note

All claims expressed in this article are solely those of the authors and do not necessarily represent those of their affiliated organizations, or those of the publisher, the editors and the reviewers. Any product that may be evaluated in this article, or claim that may be made by its manufacturer, is not guaranteed or endorsed by the publisher.
